# Roswell Park Medical Club

**Published:** 1904-06

**Authors:** George F. Cott


### SOCIETY PROCEEDINGS.

### Roswell Park Medical Club.

*Regular Meeting held May 2, 1904.*

Reported by GEORGE F. COTT, M. D., Secretary.

### NOTE ON THE USE OF IODINE AND ITS COMPOUNDS.

Dr. L. Bradley Dorr, of Buffalo, presented this subject for the consideration of the club, saying that his object was to draw attention to some experiences with the iodides and iodine in diseases and conditions not of syphilitic origin. He said: Some years ago I got a hint from a physician who was very successful in treating Bright's diseases, especially contracted kidney. He employed bichlorid of mercury and nitric acid, as the chief remedies in these diseases with a great deal of success. After using this plan of treatment for some time I substituted the protoiodide in order to obtain a greater amount of toleration by the patient than I could with the bichlorids. As these cases are for the most part chronic, it is essential that the treatment should be of a nature that can be tolerated for a long time.

I have given ¼ grain of mercury protoiodide four times a day for six months without any salivation or other inconveniences to the patient. It acts as well in acute cases of nephritis as it does in the chronic forms, and has been the most efficient remedy I have ever used in the acute cases. I have had several cases of contracted kidney in the advanced stages, accompanied with the symptoms of dropsy, shortness of breath, headaches, edema of the lungs and impaired vision. Under ordinary treatment my experience shows that these cases do not usually live more than two or three months, but with the protoiodide treatment they all have improved, the dropsy disappearing and all the other symptoms becoming so much better that the patients have resumed their duties and have lived in comparative comfort. Two cases died, one in two years after beginning treatment and one in twenty-nine months afterward, both from degeneration of the heart muscle. I have used as symptoms indicated, nitroglycerine, squills, salts, digitalis and sweat baths. In cases where the mercurial symptoms began to appear I have substituted Burnham's soluble iodine in 5-drop doses, three times a day.

### DISCUSSION.

Dr. J. C. Thompson: This subject is in line with general practice and the suggestion is of value and help in our daily work. Personally, I have had no experience with Burnham's soluble iodine, but protoiodide I have used quite extensively. I recall a case in which I gave two grains a day in eight doses of ¼ grain each for over a month, without any untoward effect. I used Parke, Davis & Co.'s preparation. You may give eight or nine tablets daily and gradually work up to the limit of toleration until the gums become sore or the teeth tender. To establish tolerance, I frequently give from three to five tablets at a time. I would like to ask Dr. Dorr if the treatment is good in slight cases of albuminuria?

Dr. J. E. KING: I wish to inquire further as regards toleration. Potassium iodide is a favorite with me; however, patients do not tolerate it very well. I use it in combination with nitroglycerine. Five grains of potassium iodide often leave a decided metallic taste and sometimes other specifics of iodine will do the same. When Burnham's soluble iodine was introduced, I used it in a case of asthma with not very encouraging results. I would like to know the toleration of soluble iodine.

Dr. THOMAS BAGLEY: Iodine is our great standby in typhoid fever. Bartholow's formula of carbolic acid and iodine certainly gives good results; symptoms all improve rapidly. I also use topical applications of the iodine to the cervix, mixed with glycerine and carbolic acid. Protoiodide of mercury in specific cases acts well. In one case I gave ¼ grain three times a day for a year and a half with complete recovery. I have also used potassium iodide in asthma with good results. In typhoid fever I give three or four drops of soluble iodine to two drops of carbolic acid three or four times a day. I would like to ask Dr. Dorr if he has used it in diabetes.

Dr. W. E. DIGNEN: Iodine is one of those drugs we use frequently, but whose results must be watched as they vary in different people. In one case I gave large doses of iodide of mercury. In a short time the teeth became loose. It was altogether the worst case of syphilis I have ever seen. I stopped the iodide for a few days and put the patient on a milder preparation. In three weeks time the ulcer healed. In a case of Bright's disease with a bad heart, the patient, having had a second attack of apoplexy, did well on soluble iodine three or four times a day. It may be given in increasing doses. Soluble iodine is a good preparation and doesn't disagree with the stomach.

Dr. T. H. MCKEE: I must confess that my experience regarding the therapeutic application of iodine is confined to surgical and specific diseases. In the former I have used it in the form of iodide of potash to combat the effects of intracranial traumata, because it is considered the proper thing to do. The patients improved, but whether their gain was due to the medication in any measure, I do not know.

Apropos of the use of protoiodide in specific cases, it is frequently next to impossible to reach and hold such patients to the physiological limit without first resorting to some more rapidly acting form of the drug, for example, the ointment. After having established the limit, they can be held with the tablets. Too often these are more or less insoluble, and that undoubtedly accounts in many cases for failure to get results. It would be of the greatest interest to know whether or not some of the striking results obtained by Dr. Dorr may not have been due to a specific condition of long standing and of which there existed unrecognisable clinical conditions. When one remembers that about one in eighteen of the general population are troubled with syphilis, it begets an attitude of suspicion toward all obscure cases, and particularly those which improve under such therapeutic measures as these under discussion.

Dr. G. F. Cott: Next to silver, iodine is the most useful drug. We must, however, ascertain the condition existing before the drug may be given with benefit. The late Dr. Pepper once wrote that a patient suspected of syphilis to whom iodide of potassium is given, and it is not tolerated, the evidence is conclusive that syphilis does not exist, but if he tolerates it he certainly has the disease. This statement aroused much controversy and the assertion was finally withdrawn. Syphilis in its different stages is a complex condition. Mercury will improve the second stage and not the third. Potassium iodide will improve the third stage and not influence the second. Iodine will not have much effect in the third stage when the body is much below par. It is sometimes difficult to select the proper combinations with iodine, for they do not act equally well.

That iodine is effective in conditions other than syphilis is amply shown. It influences glandular swellings, and mucous membranes as well. A peculiar condition is observed in testing syphilis which had not previously been treated with mercury—namely, that the iodine does not seem to exert its proper proportion of influence but will do so as soon as the body has been brought thoroughly under mercury. Schwab and Levy-Bing are now treating the newborn with soluble mercury injections of the biniodid, ½ milligram from 22,000 to 35,000 grams body weight; giving 10 to 15 injections and resting 15 days. They claim excellent results. The combination of iodine and mercury seems in many conditions to exact a more powerful influence than either drug alone.

Dr. DORR in closing said: Any kind of manufacture may be used. However, the best preparation is the tablet triturate. Protoiodide seldom causes diarrhea, but if its use is kept up it frequently stops it. I have had a case of acute nephritis with dropsy, headache and nearly total suppression of urine. Protoiodide relieved all symptoms quickly, as it also does those secreting large quantities of urine with slight albumin. In one case there was a discharge of nearly a gallon of urine a day with slight albumin; this patient also got well under iodine treatment. The patient was pregnant at the time, but took the iodine through to term. I have had no experience with iodine after labor. In polyuria, iodine is just as efficient; also in contracted kidney with little albumin.

Regarding toleration of iodine, some patients cannot stand any kind in any dose. Metallic taste in the mouth is frequent. I recall a remarkable case of iodine toleration in an asthmatic woman. I gave her four grains of potassium iodide. In a few hours an eruption of urticaria appeared. Later my father gave her an eight grain dose with a similar result, and still later I think another doctor gave her sixteen grains to the dose with a like result.

I have had the treatment of typhoid by carbolic acid brought to my attention, but am not familiar with it. As regards the physiological dose of soluble iodine, one patient took ten-drop doses without any bad effect. Dr. Marchand, of New York, has acquired a reputation all over the country for his treatment of Bright's disease with bichloride of mercury and nitric acid. Marchand's dose is: bichloride of mercury, 1-60 gr. and dilute nitric acid, three drops, three times a day. Nitric acid probably acts like nitroglycerin, by reducing the amount of albumin. I give ten drops four times a day of the standard solution of nitroglycerin.

